# Supplementary material for: Tuning instability of non-columnar neurons in the salt-and-pepper whisker map in somatosensory cortex
Source: Nat Commun. 2022 Nov 3;13:6611. doi: 10.1038/s41467-022-34261-1 (PMC9633707; doi:10.1038/s41467-022-34261-1)
Supplement: Supplementary file 1 — Supplementary Information [file 41467_2022_34261_MOESM1_ESM.pdf]

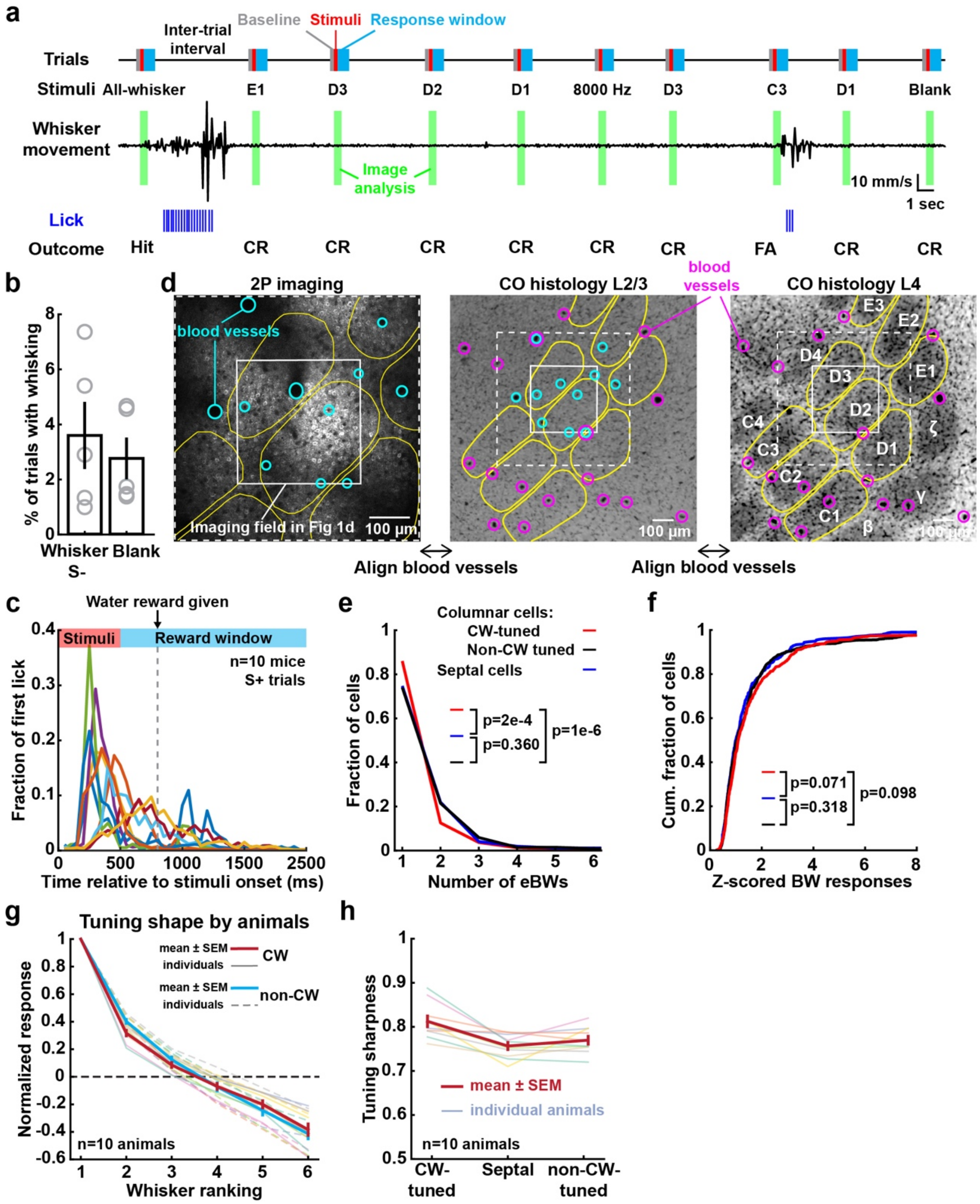

Supplementary Figure 1. Additional description of the behavioral task and imaging field localization. Related to Figure 1. (a). Contralateral whisker movement (black trace) and licking (blue raster) during an example behavioral segment containing 10 trials. Trials containing licks were not analyzed. CR: correct rejection; FA: false alarm. **(b)**. Percentage of whisker S- trials and blank trials that contained whisker movement. Each circle is one mouse. n=5 animals **(c)**. Expert mice reliably licked to S+ stimuli prior to reward delivery. Each line shows the distribution of first lick times for one mouse (averaged across imaging sessions, using 50-ms time bins). n=5 mice. **(d)**. The process of aligning the imaging field in Fig. 1d (left, solid white line) to layer 4 barrels post-hoc labeled by CO stain (right). A larger image (left, dashed line) containing the imaging field was taken at the end of experiment. Blood vessels from larger image (left, cyan circles) were aligned to CO-stained blood vessels in L2/3 (middle, cyan circles), and further aligned (magenta circles) through sequential sections to L4. **(e).**

Receptive field size quantified as the number of eBWs. Statistics: χ^2, two-sided. **(f).** The distribution of z-scored BW response of responsive neurons. **(g).** Consistency of tuning curve shape across mice. Each thin line is one mouse. CW and non-CW traces with the same color are from the same mouse. **(h).** Consistency of tuning sharpness across mice. All error bars are SEM.

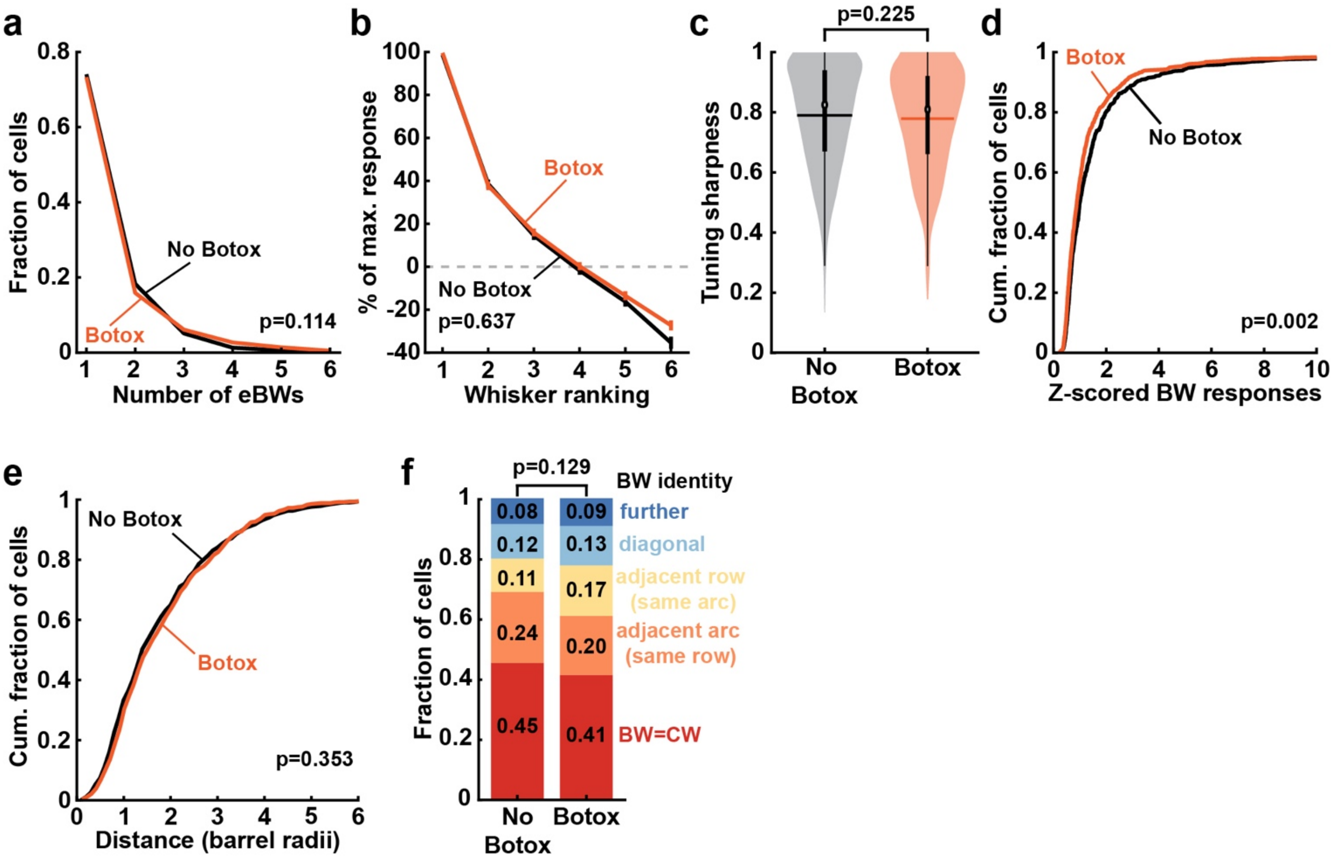

Supplementary Figure 2. No effect of Botox whisker paralysis on single cell tuning properties or whisker map topography. Related to Figures 1 and 2. Comparison of single cell responses to whisker stimuli in 4 mice before and after Botox treatment. Before Botox: n=16 imaging fields, 841 whisker-responsive cells. After Botox: n=16 imaging fields, 767 whisker-responsive cells. **(a)**. Receptive field size quantified as the number of eBWs. Statistics: χ^2, two-sided. **(b).** Mean within-cell rank ordered tuning curve, after normalizing response magnitudes to maximal response of the cells. Statistics: unbalanced two-way ANOVA. **(c).** Distribution of mean tuning sharpness of responsive neurons. Circles are medians, and horizontal lines are means. Statistics: rank-sum, two-sided. **(d).** The distribution of z-scored BW response of responsive neurons. Statistics: KS, two-sided. **(e).** Distance to BW column center for responsive cells. Statistics: KS, two-sided. **(f).** Identity of BW for all responsive cells in a column. Statistics: χ^2, two-sided. All error bars are SEM.

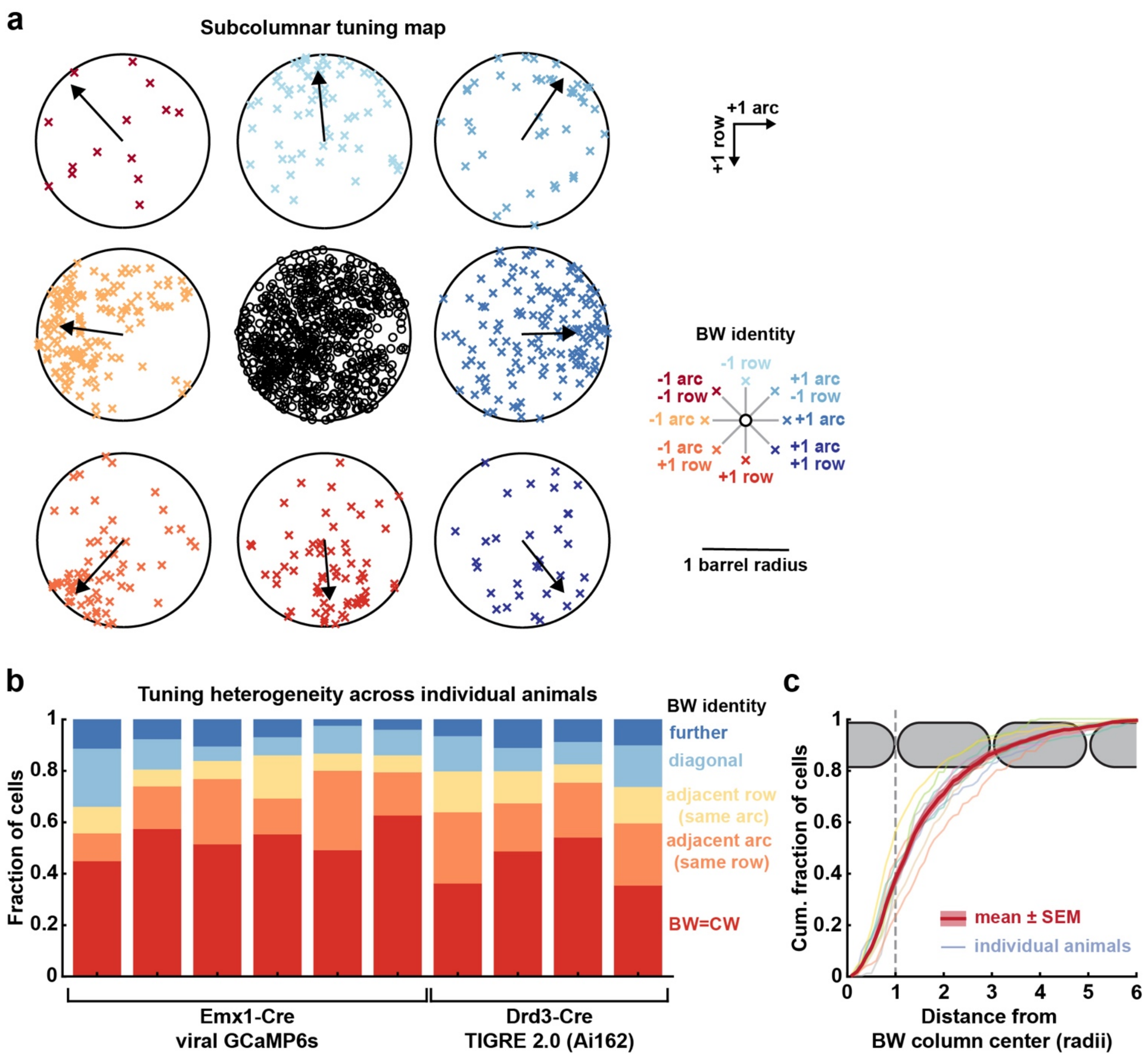

Supplementary Figure 3. Additional characterization of whisker map organization for L2/3 PYR neurons. Related to Figure 2. (a). Subcolumnar location of PYR neurons tuned to different whiskers in a reference column. Cells tuned to different whiskers are intermixed (as in Fig. 2a), but are shown separated here so that their subcolumnar position can be seen. The black circle is the border of the reference whisker column. Arrows show circular mean of cell positions. **(b).** Consistency of tuning heterogeneity across mice. For each mouse, the fraction of cells tuned to different whiskers, relative to the columnar whisker, is shown. **(c).** Consistency of cell location relative to BW column center, across mice. Each thin line is cumulative distribution of cell location for one mouse.

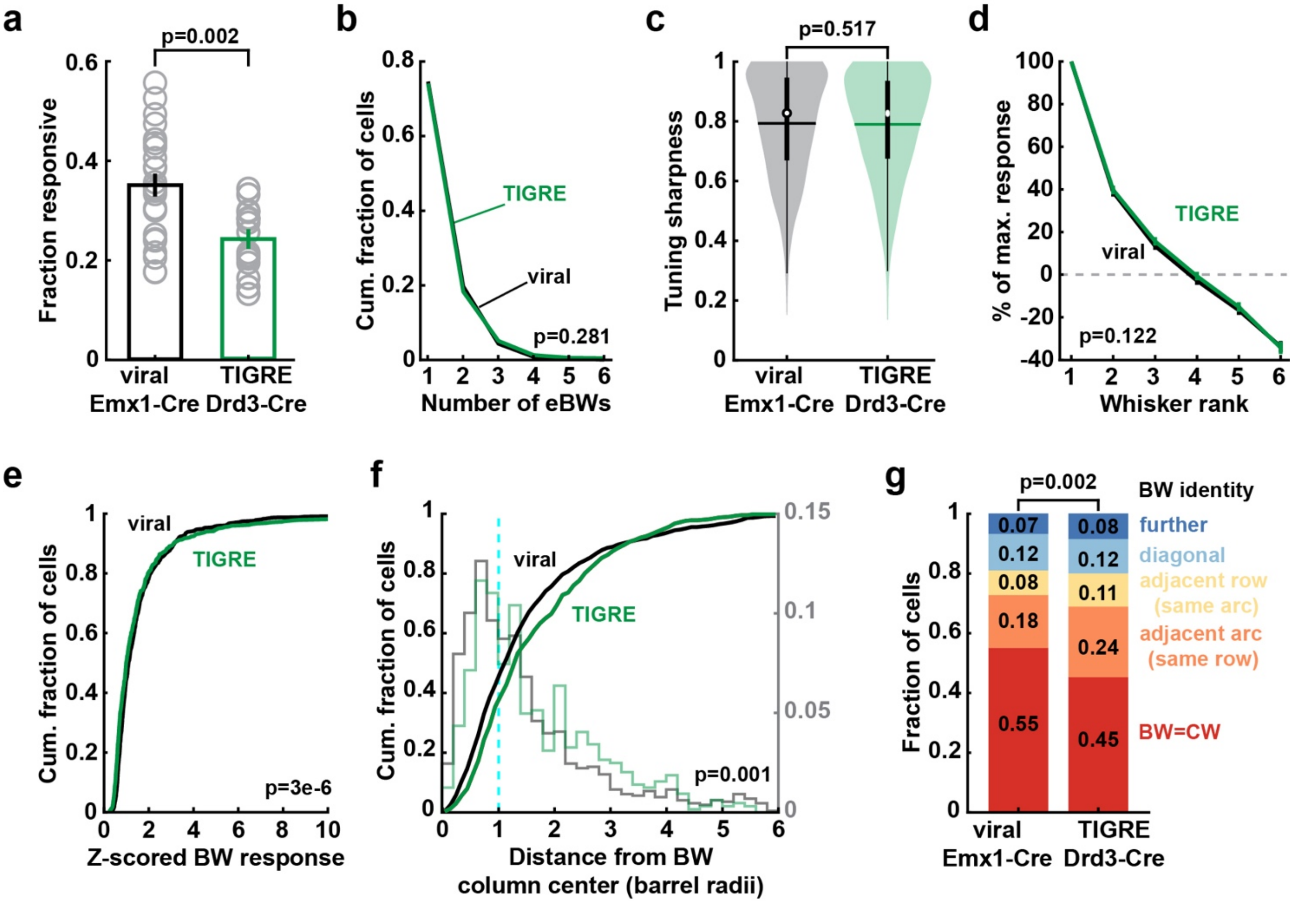

Supplementary Figure 4. Emx1-Cre and Drd3-Cre mice have similar single cell tuning but slightly different tuning heterogeneity. Related to Figures 1 and 2. (a). Fraction of whisker-responsive PYR neurons per imaging field for mice with virally expressed GCaMP6s (Emx1-Cre mutants injected with AAV1-Syn-Flex-GCaMP6s-WPRE-SV40, n=24 fields) and mice with transgenically expressed GCaMP6s (Drd3-Cre:TIGRE2.0, n=16 fields). Each circle is one imaging field. Statistics: rank-sum, two-sided. Error bars: SEM. **(b)**. Receptive field size quantified as the number of eBWs. Statistics: χ^2, two-sided. **(c)**. Distribution of mean tuning sharpness of responsive neurons. Circles are medians, and horizontal lines are means. Thick vertical lines show interquartile range and thin vertical lines are 1.5 x interquartile range. Statistics: rank-sum, two-sided. **(d)**. Mean within-cell rank ordered tuning curve, after normalizing response magnitudes to each cell's maximal response. Error bars: SEM. Statistics: unbalanced two-way ANOVA. **(e)**. Distribution of z-scored BW response of responsive neurons. Statistics: KS, two-sided. **(f)**. Cumulative and binned distribution of distance from responsive cells to their BW column center. Statistics: KS, two-sided. **(g)**. Identity of BW for all responsive cells in a column. Statistics: χ^2, two-sided. (b-g): n=1387 cells in virally expressed GCaMP6s mice and n=841 cells in transgenically expressed GCaMP6s mice.

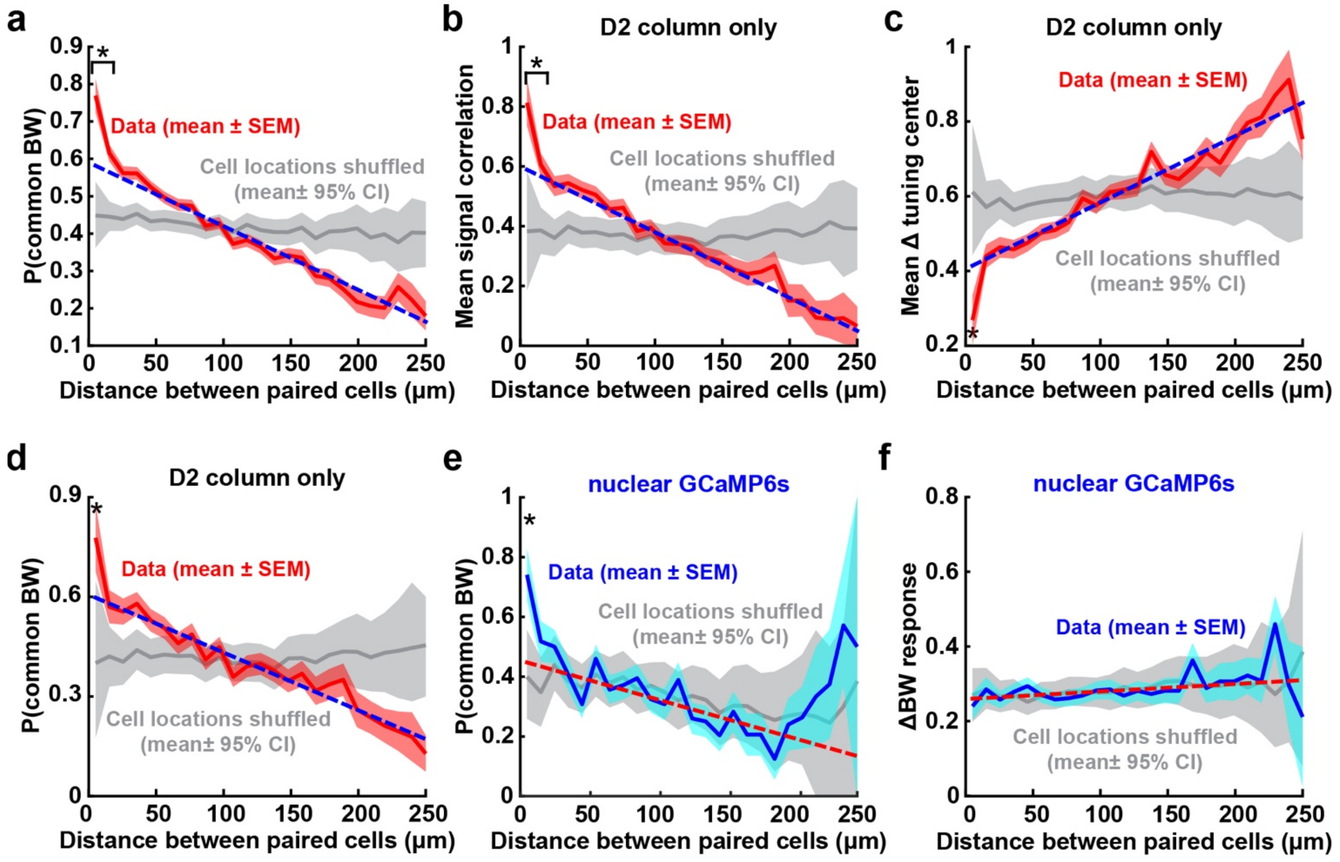

Supplementary Figure 5. Additional analysis of local tuning clusters in whisker columns. Related to Figure 3. **(a)**. Probability of sharing the same BW, for pairs of co-columnar PYR neurons. Red: observed data. Gray: spatially shuffled neurons. Dashed line: linear regression of measured data from 30 to 200 μm. **(b-d)** Analysis of tuning clusters using only cells within anatomical D2 columns. Convention as in (a). **(b).** Mean signal correlation for pairs of neurons. **(c).** Mean difference in tuning center-of-mass. **(d).** Probability of sharing the same BW. **(e-f)** Analysis of tuning clusters using nucleus localized H2B-GCaMP6s. Conventions as in (a-d). Asterisks indicate significant difference from extrapolated linear regression by one-sided permutation test within each bin and corrected for multiple comparisons with false discovery rate 0.05. (see Methods).

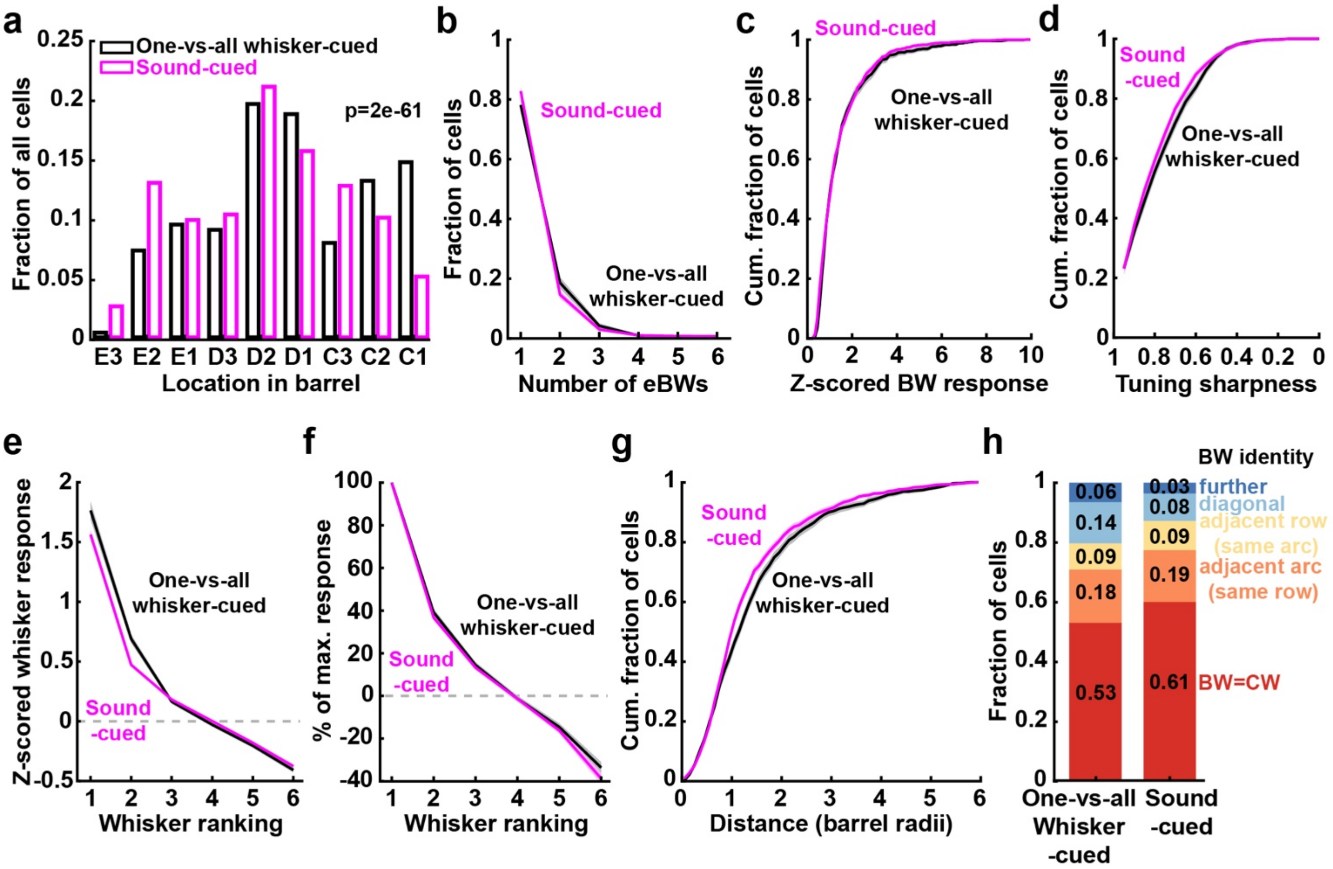

Supplementary Figure 6. Comparison of whisker tuning and maps in whisker-cued and sound-cued mice, after sub-sampling to match spatial distribution of neurons. Related to Figure 4. (a). Anatomical columnar locations of all imaged PYR neurons, showing modest mismatch between whisker-cued and sound-cued populations. Statistics: χ^2, two-sided. **(b-h)**. Functional properties of PYR neurons in whisker-cued and sound-cued mice compared after spatial sub-sampling to achieve exactly matched columnar distribution. See Materials and Methods. **(b)**. Receptive field size quantified as the number of eBWs. **(c)**. Mean BW response magnitude, after normalizing response magnitudes to blank responses. **(d)**. Distribution of mean tuning sharpness of responsive neurons **(e)**. Mean within-cell rank ordered tuning curve of neurons. **(f)**. Mean within-cell rank ordered tuning curve, after normalizing response magnitudes to maximal response of the cells. **(g)**. Cumulative distribution of distance from responsive PYR neurons to their BW column center. **(h)**. Identity of BW for all responsive cells in a column. All lines and error bars or shading show mean and 95% confidence interval, respectively, from sub-sampling to ensure matched spatial cell distributions.

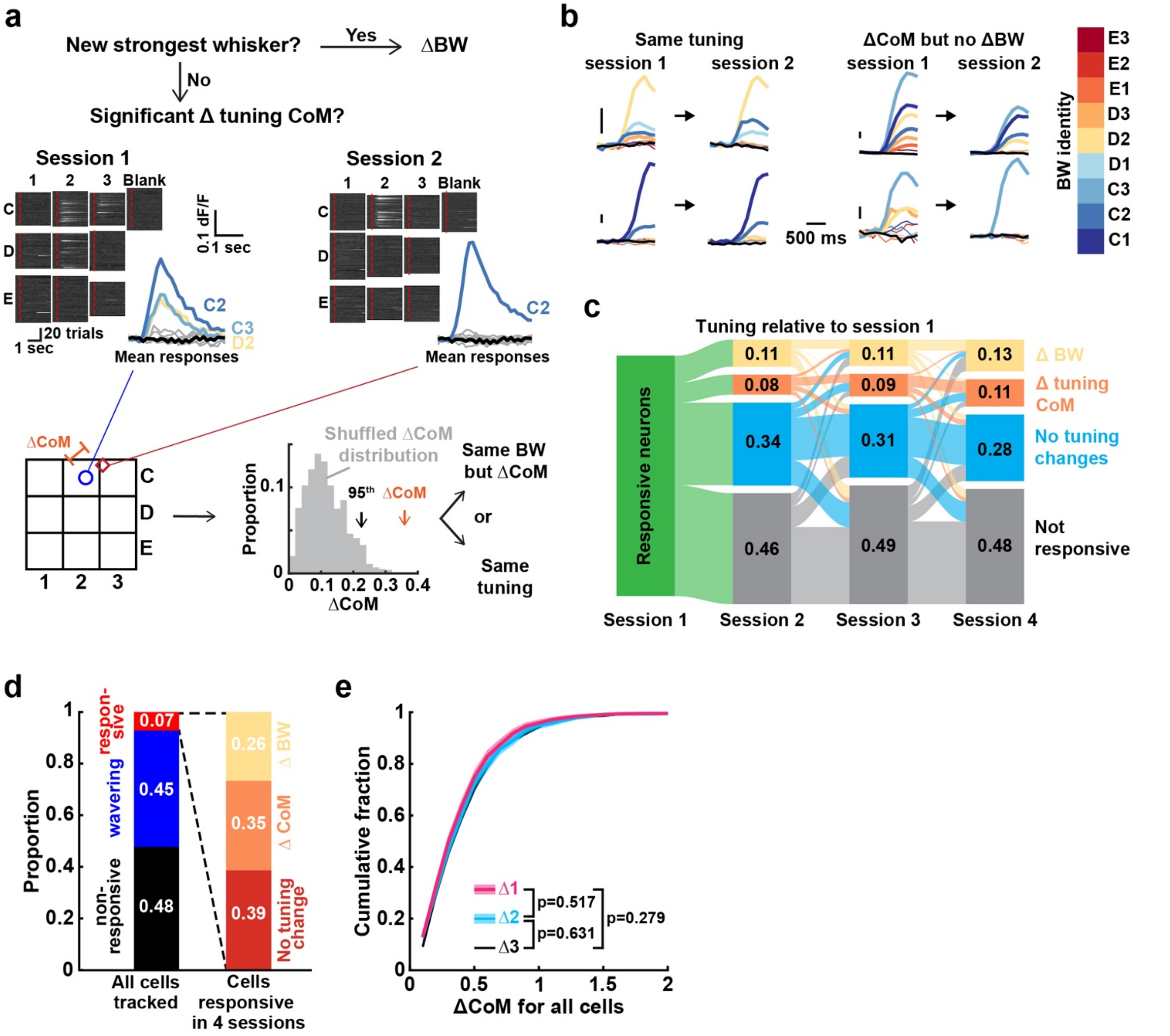

Supplementary Figure 7. Measurement of tuning instability via longitudinal Ca^{2+} imaging. Related to Figure 5. (a). Method to test for changes in tuning center-of-mass (ΔCoM) across sessions. See Methods for statistics. Example shows a cell whose BW is C2 in both sessions, but whose CoM changes significantly relative to the null model simulated by permuting trials across sessions (gray shading). **(b).** Additional example cells showing either stable tuning across 2 sessions (left) or a significant change in CoM but no change in BW (right). Each trace is mean whisker-evoked ΔF/F for one whisker (colors) or for blank trials (black). Scale bar: 0.1 ΔF/F. **(c).** Changes in tuning for neurons which were responsive in all four sessions. **(d)**. Stability of responsiveness and tuning for the subset of neurons that were successfully imaged over all 4 sessions (n=2771 cells, 4 mice). Among neurons that were responsive in all 4 sessions, tuning was scored as unstable if a significant change occurred between any two sessions. **(e).** Cumulative distribution of ΔCoM for all neurons across Δ1, Δ2 or Δ3 intervals. Statistics: KS, two-sided.

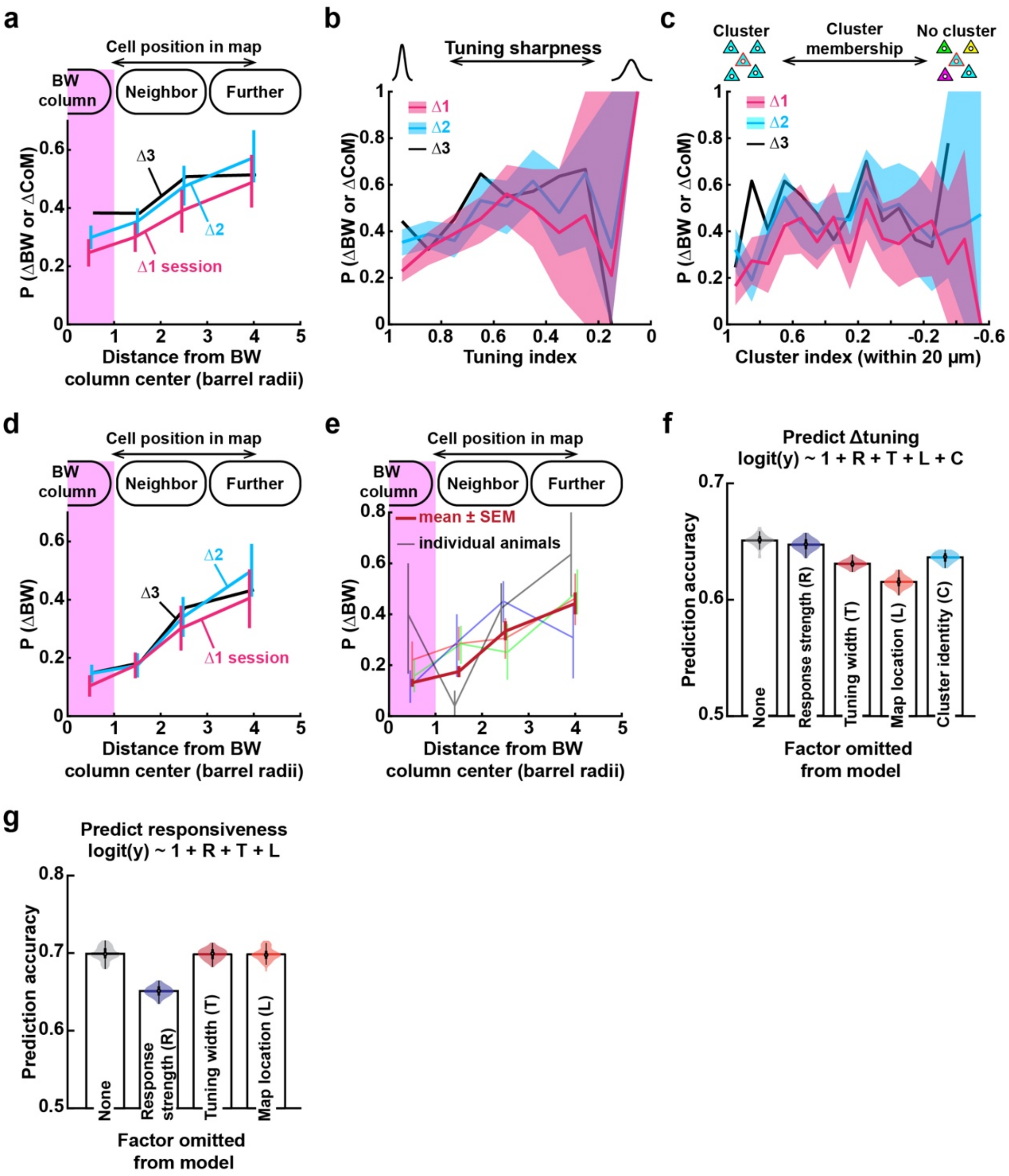

Supplementary Figure 8. Additional analysis of tuning instability within the L2/3 whisker map. Related to Figure 6. (a). Spatial organization of tuning changes (either ΔBW or ΔCoM) calculated separately for Δ1 (n=473 pairs), Δ2 (n=408 pairs) and Δ3 (n=389 pairs) intervals. Same dataset used in (b-d). **(b)**. Relationship between probability of tuning change and initial tuning sharpness, calculated separately for Δ1, Δ2 and Δ3 intervals. **(c)**. Relationship between probability of tuning change and cluster index in session 1, calculated separately for Δ1, Δ2 and Δ3 intervals. **(d)**. Spatial organization of tuning changes, showing only cells with significant ΔBW, calculated separately across Δ1, Δ2 and Δ3 intervals. Conventions as in (a). **(e)**. Consistency of spatial organization of BW changes across mice. Each thin line is one mouse. n=4 animals. **(f).** Analysis of which of 4 factors in the first session predict whether a cell will change its tuning in the second session (n=1270 pairs of data), using a generalized linear model, with 10-fold cross-validation. Once the model is trained, we assessed the decrement in model performance as individual factors are excluded. Results show that map location was the strongest single factor predicting tuning change. **(g)**. Similar analysis to predict whether a neuron changes responsiveness. Response strength in the first session was the strongest single factor predicting responsiveness change. Error bars and shading in (a-d) are bootstrapped 95% CI after subsampling to ensure each cell is represented only once. Violin plots in (f-g) are results from cross-validated model runs.

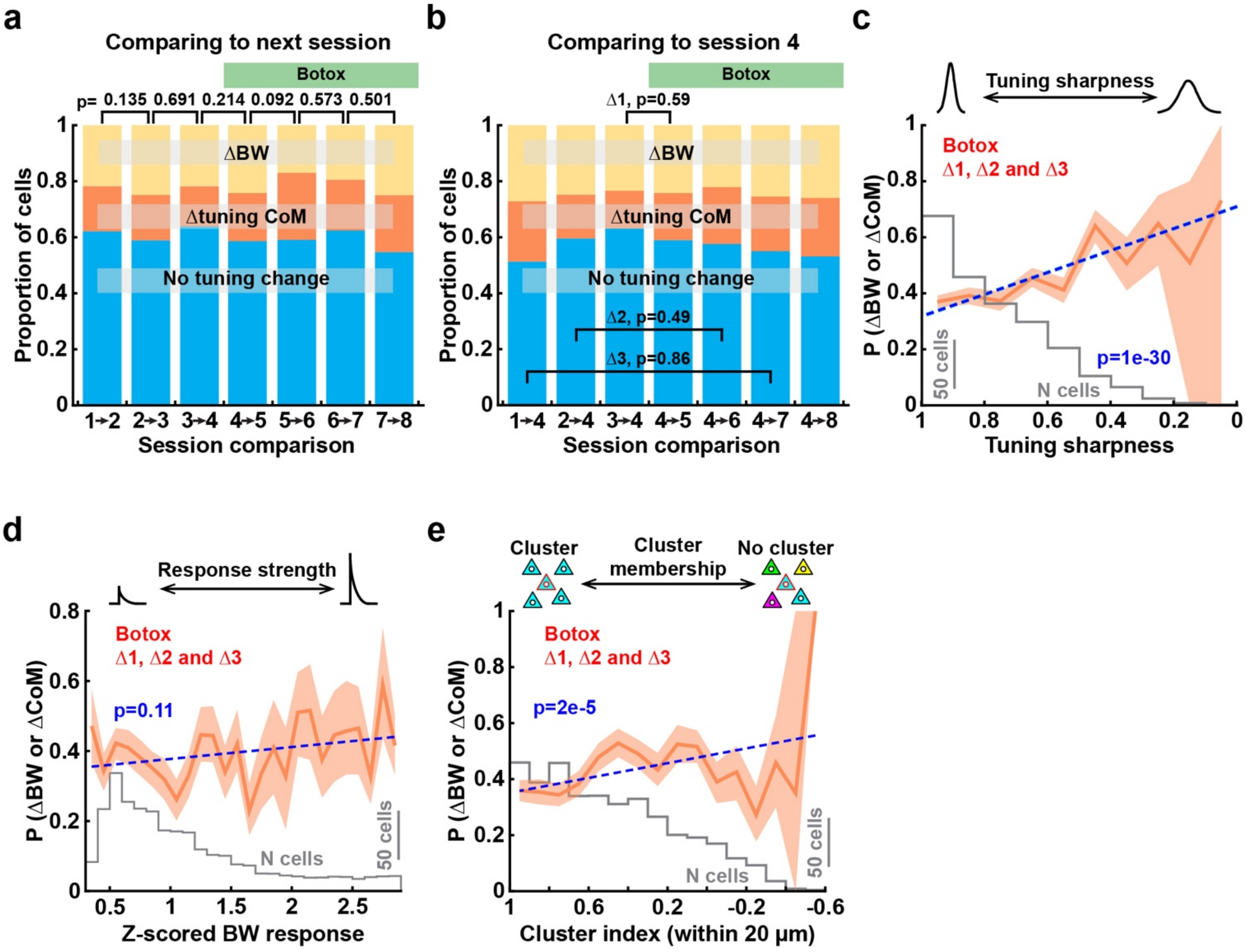

Supplementary Figure 9. Disrupting whisking does not affect tuning instability. Related to Figure 7. (a). Fraction of PYR cells with significant tuning changes between each adjacent imaging session. Only cells that were whisker-responsive across all 8 sessions were included. The proportion of cells with tuning instability did not differ between pre-Botox sessions and post-Botox sessions (statistics: χ^2, two-sided). **(b)**. Same as (a), but testing for significant tuning changes in each session relative to Session 4, which is the session immediately before Botox treatment. The proportion of cells with tuning instability at Δ1, Δ2 or Δ3 intervals was not different between pre-Botox vs. post-Botox epochs (statistics: χ^2, two-sided). **(c)**. Probability of tuning change as a function of initial tuning sharpness, pooled over Δ1, Δ2 and Δ3 Botox sessions. Dashed line: linear regression. Statistics: two-sided t-statistic for slope ≠ zero. **(d)**. Same as (c), but as a function of initial responsiveness to the BW. **(e)**. Same as (c), but as a function of the cluster index. For (c-e), lines and shadings represent mean and bootstrapped 95% confidence interval after subsampling to ensure each cell is represented once in the dataset.

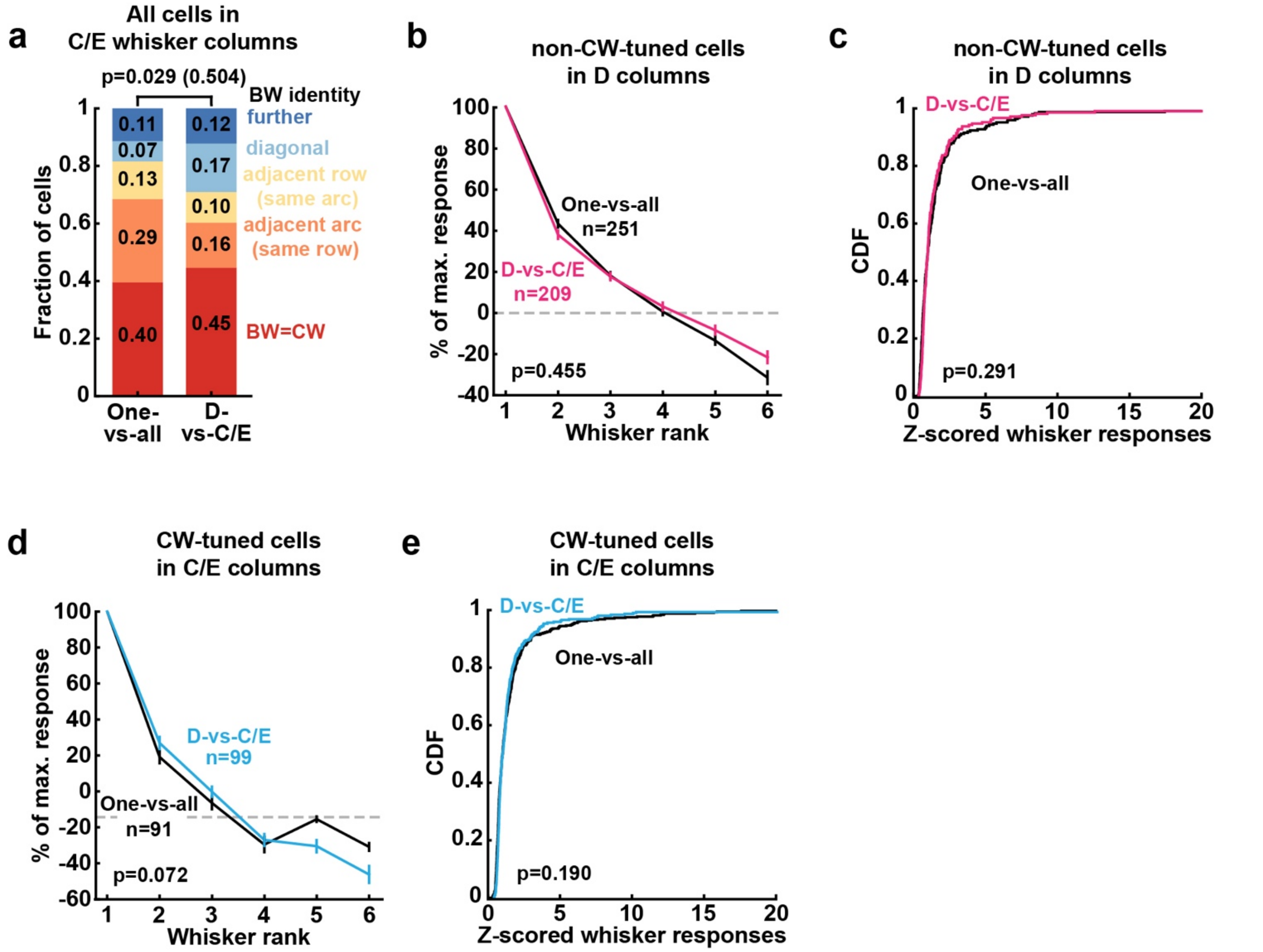

Supplementary Figure 10. Additional analysis of tuning instability in D-versus-C/E discrimination expert mice. Related to Figure 8. (a). Tuning heterogeneity for cells in C- or E-row columns, compared between one-versus-all mice (n=91 cells from 4 mice) and D-versus-C/E mice (n=99 cells from 2 mice). Statistics: two-sided χ^2 for difference in distribution, and (in parentheses) Fisher's exact test for proportion of CW-tuned cells. **(b).** Mean rank-ordered whisker tuning curves for non-CW-tuned neurons in D whisker columns. *p*-value is for behavior factor in unbalanced two-way ANOVA. **(c).** CW response magnitude for the same cells as in (b). Statistics: KS. **(d).** Mean rank-ordered whisker tuning curves for CW-tuned neurons in C or E row columns. Conventions as in (b). **(e).** BW response magnitude for CW-tuned neurons in C or E row columns. Statistics: KS, two-sided. Conventions as in (c). All error bars are SEM.
