# Supplementary material for: Tuning instability of non-columnar neurons in the salt-and-pepper whisker map in somatosensory cortex
Source: Nat Commun. 2022 Nov 3;13:6611. doi: 10.1038/s41467-022-34261-1 (PMC9633707; doi:10.1038/s41467-022-34261-1)
Supplement: Supplementary file 3 — Reporting Summary [file 41467_2022_34261_MOESM3_ESM.pdf]

nature portfolio

Corresponding author(s): Daniel E. Feldman

Last updated by author(s): Oct 11, 2022

Reporting Summary

Nature Portfolio wishes to improve the reproducibility of the work that we publish. This form provides structure for consistency and transparency in reporting. For further information on Nature Portfolio policies, see our Editorial Policies and the Editorial Policy Checklist.

Statistics

For all statistical analyses, confirm that the following items are present in the figure legend, table legend, main text, or Methods section.

n/a	Confirmed	
☐	☒	The exact sample size (*n*) for each experimental group/condition, given as a discrete number and unit of measurement
☐	☒	A statement on whether measurements were taken from distinct samples or whether the same sample was measured repeatedly
☐	☒	The statistical test(s) used AND whether they are one- or two-sided<br>*Only common tests should be described solely by name; describe more complex techniques in the Methods section.*
☐	☒	A description of all covariates tested
☐	☒	A description of any assumptions or corrections, such as tests of normality and adjustment for multiple comparisons
☐	☒	A full description of the statistical parameters including central tendency (e.g. means) or other basic estimates (e.g. regression coefficient) AND variation (e.g. standard deviation) or associated estimates of uncertainty (e.g. confidence intervals)
☐	☒	For null hypothesis testing, the test statistic (e.g. *F*, *t*, *r*) with confidence intervals, effect sizes, degrees of freedom and *P* value noted<br>*Give P values as exact values whenever suitable.*
☒	☐	For Bayesian analysis, information on the choice of priors and Markov chain Monte Carlo settings
☒	☐	For hierarchical and complex designs, identification of the appropriate level for tests and full reporting of outcomes
☒	☐	Estimates of effect sizes (e.g. Cohen's *d*, Pearson's *r*), indicating how they were calculated

Our web collection on statistics for biologists contains articles on many of the points above.

Software and code

Policy information about availability of computer code

Data collection	Behavioral control and stimulus delivery used custom software programmed in Igor Pro 6.0 (Wavemetrics). Data collection was performed using ScanImage 5.6 (Vidrio Technologies) under the MatLab R2019b environment (MathWorks)
Data analysis	Imaging analysis and statistical tests used CaImAn (https://github.com/flatironinstitute/CaImAn- MATLAB), NoRMCorre (https://github.com/ flatironinstitute/NoRMCorre) and custom software in MatLab R2019b (MathWorks).

For manuscripts utilizing custom algorithms or software that are central to the research but not yet described in published literature, software must be made available to editors and reviewers. We strongly encourage code deposition in a community repository (e.g. GitHub). See the Nature Portfolio guidelines for submitting code & software for further information.

Data

Policy information about availability of data

All manuscripts must include a data availability statement. This statement should provide the following information, where applicable:

- Accession codes, unique identifiers, or web links for publicly available datasets
- A description of any restrictions on data availability
- For clinical datasets or third party data, please ensure that the statement adheres to our policy

Imaging data and analysis code will be available upon publication in the Feldman lab GitHub repository, https://github.com/dfeldman189/PublicData

Field-specific reporting

Please select the one below that is the best fit for your research. If you are not sure, read the appropriate sections before making your selection.

☒ Life sciences ☐ Behavioural & social sciences ☐ Ecological, evolutionary & environmental sciences

For a reference copy of the document with all sections, see nature.com/documents/nr-reporting-summary-flat.pdf

Life sciences study design

All studies must disclose on these points even when the disclosure is negative.

Sample size	We did not perform a priori sample-size estimation. Instead, sample size was planned to match standard practice in Ca2+ imaging studies of population coding, for number of animals, number of imaging fields, and total number of neurons (e.g., Neuron. 2015 Jun 17;86(6):1478-90, Nature. 2013 Jul 18;499(7458):336-40, Nat Neurosci. 2012 Nov;15(11):1539-46).
Data exclusions	Only ROIs located in or near one of the 9 stimulated whisker columns were analyzed (≤ 1.25 barrel radii from the centroid of one of these columns). This ensured that relevant whisker stimuli were presented to map receptive fields. Two mice with viral GCaMP injection were excluded before imaging began, due to bone regrowth and thickened dura that prevented effective imaging.
Replication	Salt-and-pepper map organization was replicated in all individual mice, for both viral and transgenic expression methods (Suppl. Figs. 3 and 4). Tuning instability was replicated in each longitudinally imaged mouse (Supp. Fig. 8e). The sound-cued experiment (Fig. 4) was performed in 5 mice, trained as 2 separate cohorts, and all 5 mice (both cohorts) showed similar results, with robustness of this result established by a data resampling procedure (Suppl. Fig. 6)
Randomization	Assignment to WC and SC groups was not randomized, but were run as two sequential cohorts of mice. Both groups were on the same strain background, received the same AAV injection, and were presented the identical stimulus set. The main covariate that could impact results was location of imaged neurons, which we controlled by a data resampling procedure (Suppl. Fig. 6).
Blinding	Blinding during data collection for the type of behavior or Botox injection status is not possible. But for image analysis and ROI curation, the investigator was blind to any stimulus- or location-related information. The remaining analysis was all automated and equivalent across groups.

Reporting for specific materials, systems and methods

We require information from authors about some types of materials, experimental systems and methods used in many studies. Here, indicate whether each material, system or method listed is relevant to your study. If you are not sure if a list item applies to your research, read the appropriate section before selecting a response.

Materials & experimental systems

n/a	Involved in the study	
☒	☐	Antibodies
☒	☐	Eukaryotic cell lines
☒	☐	Palaeontology and archaeology
☐	☒	Animals and other organisms
☒	☐	Human research participants
☒	☐	Clinical data
☒	☐	Dual use research of concern

Methods

n/a	Involved in the study	
☒	☐	ChIP-seq
☒	☐	Flow cytometry
☒	☐	MRI-based neuroimaging

Animals and other organisms

Policy information about studies involving animals; ARRIVE guidelines recommended for reporting animal research

Laboratory animals	Mouse (Mus muscularis) transgenic strains were used. Drd3-Cre mice (MMRRC_034610-UCD) and TIGRE2.0 (Ai162, JAX 031562) mice were on mixed background. Emx1-IRES-Cre (JAX 005628) mice were on C57BL/6J background. Additional C57BL/6J mice were used for the nucleus-targeted GCaMP6s experiment. Mice were 2.5-3 months old at surgery. Viral-GCaMP6s mice were 5 males, 8 females, and Drd3-Cre:TIGRE2.0 mice were 2 males, 4 females. Mice were on a 12:12 light cycle and imaged during the dark phase.
Wild animals	No wild animals were used.
Field-collected samples	No field collection
Ethics oversight	Animal procedures were approved by the UC Berkeley Animal Care and Use Committee, and followed NIH guidelines

Note that full information on the approval of the study protocol must also be provided in the manuscript.
